# Shell Metal Profiles of Caspian Bivalves Show Genus-Specific Patterns with Potential Relevance for Biomonitoring in the Southern Caspian Sea

**DOI:** 10.3390/ani16101491

**Published:** 2026-05-13

**Authors:** Shima Bakhshalizadeh, Rafael Mora-Medina, Nahúm Ayala-Soldado

**Affiliations:** 1 Department of Marine Science, Caspian Sea Basin Research Center, University of Guilan, Rasht 41996-13776, Iran; sh.bakhshalizadeh@guilan.ac.ir; 2 Department of Anatomy and Comparative Pathology and Toxicology, Faculty of Veterinary Medicine, University of Córdoba, 14014 Córdoba, Spain

**Keywords:** bivalves, shell geochemistry, trace elements, relative multielement loading, Caspian Sea, biomonitoring

## Abstract

Bivalves are widely used in aquatic monitoring, but the potential of their shells to provide comparative metal signatures remains insufficiently explored in the Caspian Sea. This study quantified major and trace elements in empty shells of five bivalve genera (*Cerastoderma*, *Didacna*, *Dreissena*, *Hypanis*, and *Mytilaster*) collected from a single shell accumulation site on the southern Caspian coast. The aim was to assess intergeneric variability and provide a preliminary comparative framework for shell-based metal profiling. Element concentrations were measured by inductively coupled plasma mass spectrometry (ICP-MS), and median concentrations, internal residual enrichment factors, relative concentration factors, a relative multielement loading index, and centered log-ratio principal component analysis were applied as within-dataset comparative tools. Significant differences among genera were found for most elements. *Dreissena* and *Cerastoderma* showed the highest relative multielement loading, with comparatively elevated values of Cr, Fe, and Hg, whereas *Hypanis* showed marked element-specific deviations, particularly for Cu and Co. In contrast, *Mytilaster* and *Didacna* generally showed lower overall relative loading, although *Mytilaster* also displayed a strong Co-specific pattern. PCA-clr analysis showed structured genus-related separation based on multielement shell composition, with contrasting associations among Al, As, Cr, Fe, Co, Cu, Hg, and carbonate- or shell-associated elements such as Ba, Mg, Sr, Ti, and Zn. Overall, the results indicate that Caspian bivalve shells display distinct genus-related multielement profiles. Within the limitations of a single-site shell-accumulation dataset and the absence of paired environmental, tissue, mineralogical, and microstructural data, these findings should be interpreted as a preliminary comparative assessment rather than as evidence of environmental contamination or validated bioindicator performance. The results identify genera and elements that may deserve priority in future shell-based biomonitoring studies after validation with living populations, broader spatial replication, and paired sediment, water, particulate, and soft-tissue data.

## Simple Summary

Metal contamination is a long-term concern in coastal ecosystems because metals can persist in the environment and become incorporated into biological materials over time. In semi-enclosed systems such as the Caspian Sea, there is a need for complementary tools to compare metal-related signatures in coastal organisms. In this study, we analyzed empty shells of five bivalve genera collected from a single shell accumulation site on the southern Caspian coast. Only adult, visibly well-preserved shells without visible abrasion were selected to improve comparability among specimens. The results showed that shell metal composition differed clearly among genera. *Dreissena* and *Cerastoderma* showed the highest relative multielement loading, whereas *Mytilaster* and *Didacna* generally showed lower overall loading. *Hypanis* was characterized by marked element-specific patterns, particularly for Cu and Co. These findings suggest that bivalve shells can provide useful comparative information on genus-related metal profiles in the Caspian Sea. However, validation with living populations, replicated sampling sites, and paired sediment, water, particulate, and soft-tissue data will be necessary before these shells can be used as reliable monitoring indicators.

## 1. Introduction

Metal contamination is one of the most persistent and widespread pressures affecting aquatic ecosystems and the organisms that inhabit them. Because metals are non-degradable and can persist in water, sediments, and biological tissues, they represent long-term chemical stressors with important ecological and human health implications [1]. These elements enter aquatic environments through multiple pathways, including riverine discharge, industrial effluents, atmospheric deposition, and sediment resuspension, where they may remain in dissolved form or become associated with fine particles and organic matter [2,3]. Unlike many organic pollutants, metals are not broken down into harmless end products but are instead transported, transformed, and stored within aquatic systems, leading to chronic exposure and accumulation in aquatic organisms [4].

The Caspian Sea, the largest enclosed inland water body on Earth, is particularly vulnerable to metal accumulation because of its restricted water exchange, high sedimentation rates, and intense anthropogenic pressure along its coastline [5]. Industrial activities, including oil-related operations, together with riverine and coastal anthropogenic inputs, contribute to trace metal contamination in Caspian coastal sediments and benthic environments [6]. Because water renewal is limited in this semi-enclosed basin, contaminants can be retained for long periods in sediments and aquatic organisms, increasing the need for reliable monitoring tools. Evidence of elevated metal concentrations in southern Caspian coastal sediments further highlights the need for complementary biological and geochemical approaches able to describe metal-related patterns in coastal environments [7].

Bivalves are widely used in aquatic monitoring because their sedentary lifestyle, broad distribution, and filter-feeding activity allow them to reflect local chemical conditions over time [8]. Their close interaction with suspended particles, bottom sediments, and dissolved substances makes them useful organisms for studying metal exposure in coastal environments. Their shells, composed mainly of calcium carbonate and organic matrix components, can incorporate trace elements during biomineralization under both environmental and physiological influence. This means that shell chemistry may preserve information not only about element availability in the surrounding environment but also about biologically mediated differences in elemental incorporation among taxa [9]. As a result, shells can provide a durable matrix for comparing trace-element profiles among organisms, locations, or time periods, and previous studies have shown that shell elemental fingerprints may help discriminate environmental origin or harvesting location [10]. Compared with soft tissues, which are more strongly affected by short-term physiological variation and faster turnover, shells may retain longer-term chemical information and therefore complement tissue-based approaches in retrospective biomonitoring [11].

Shell-based biomonitoring has emerged as a cost-effective and ecologically relevant approach for assessing metal-related patterns in marine and estuarine systems. Previous studies in carbonate-producing organisms, including mussels, oysters, barnacles, and foraminifera, have shown that biogenic hard parts can record trace-element variation linked to both natural processes and anthropogenic inputs [12,13]. Because shells are durable and can persist long after the death of the organism, they offer opportunities for retrospective comparisons across locations and time periods. In addition, advances in analytical and multivariate methods have improved the capacity to describe multielement shell signatures and relate them to environmental or biological gradients [10]. However, for the Caspian Sea, shell-based metal profiling remains insufficiently explored, particularly from a comparative, multi-genus perspective. Much of the available evidence on metal contamination in the region is based on sediments, water, or soft tissues, whereas the extent to which bivalve shells from Caspian coastal deposits retain genus-related multielement signatures remains unclear. This represents a relevant gap because shell accumulation deposits are common and accessible archives, but their interpretation is complicated by taxonomic differences, postmortem history, surface-associated material, and the absence of paired environmental data.

In the present study, we quantified major and trace elements in empty shells of five bivalve genera (*Cerastoderma*, *Didacna*, *Dreissena*, *Hypanis*, and *Mytilaster*) collected from a single shell accumulation site on the southern Caspian coast. By combining concentration data with internal residual enrichment factors (EFs), relative concentration factors (RCFs), the relative multielement loading index (MPI), and principal component analysis on centered log-ratio transformed data (PCA-clr), we aimed to describe intergeneric variability in shell metal profiles, identify element–genus combinations that may deserve priority in future validation studies, and provide a preliminary comparative framework for shell-based metal profiling in the Caspian Sea.

## 2. Materials and Methods

### 2.1. Sample Location

**Figure 1:**
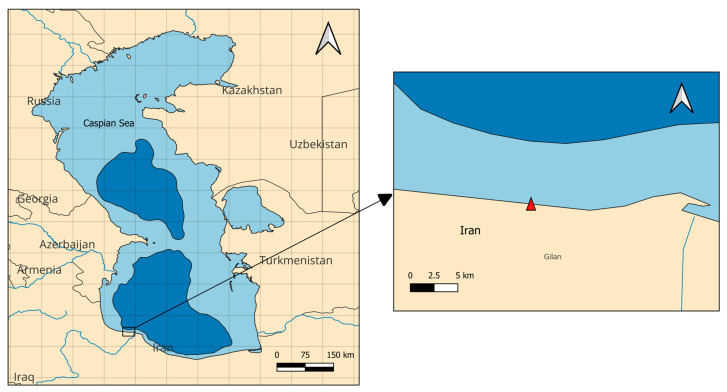
Location of the sampling site, indicated by a red triangle, along the southern Caspian Sea coast, Iran.

A total of 48 bivalve shells representing five genera (*Hypanis*, *Mytilaster*, *Dreissena*, *Didacna*, and *Cerastoderma*) were collected in autumn 2020 from a shell accumulation site on the coast of Gilan Province, in the southwestern Caspian Sea, Iran (Figure 1). The analyzed material comprised 11 *Cerastoderma*, 9 *Hypanis*, 9 *Didacna*, 9 *Dreissena*, and 10 *Mytilaster* shells. Shells were not selected at random from the accumulation deposit; instead, only adult, well-preserved shells without visible abrasion or marked surface deterioration were included in order to improve comparability among specimens. Within each genus, shells with a comparable number of external growth rings were preferentially selected. Taxonomic identification was performed to the genus level using the Atlas of Caspian Sea Invertebrates [14]. Genus-level identification was retained as the most conservative and reproducible taxonomic resolution for the available shell material, avoiding unsupported species-level assignments based only on shell morphology. Before analysis, shells were brushed to remove adhering material, rinsed thoroughly with Milli-Q deionized water (Merck KGaA, Darmstadt, Germany) and air-dried at room temperature under a laminar flow hood. Shell size and weight were recorded, and external growth rings were counted as a comparative descriptor of shell age. Cleaned and dried shells were stored individually in clean, sealed polyethylene containers at room temperature in a dry, dust-free environment until chemical analysis.

### 2.2. Chemical Elements Analysis

Bivalve shell samples were processed for the determination of trace and major element concentrations. After drying, each shell was individually crushed and ground to a fine powder using a non-metallic grinding system. The resulting powder was thoroughly mixed to obtain a representative homogenized sample for each shell, and the grinding equipment was carefully cleaned between samples to minimize cross-contamination. Approximately 0.5 g of homogenized shell powder was digested with 10 mL of ultrapure nitric acid (HNO_3_, 65%, Suprapur®, Merck KGaA, Darmstadt, Germany) using a closed-vessel microwave digestion system (MARS 6, CEM Corporation, Matthews, NC, USA). The digestion program consisted of a gradual temperature increase to 180 °C over 15 min, followed by a 20 min holding step. After cooling, digested samples were quantitatively transferred and diluted to a final volume of 25 mL with Milli-Q water (18.2 MΩ·cm). Aliquots of the digested samples were further diluted, when required, to match the calibration matrix and to bring major elements within the linear calibration range. Solutions were stored in acid-washed high-density polyethylene bottles until analysis.

Elemental quantification was performed using an Agilent 7500 series inductively coupled plasma mass spectrometer (ICP-MS; Agilent Technologies Inc., Santa Clara, CA, USA). The analytical suite included Al, As, Ba, Ca, Cd, Co, Cr, Cu, Fe, Hg, Mg, Mn, Ni, Pb, Sr, Ti, V, and Zn. External calibration was performed using certified ICP-MS calibration standards from Agilent Technologies Inc. (Santa Clara, CA, USA). Al, As, Ba, Ca, Cd, Co, Cr, Cu, Fe, Mg, Mn, Ni, Pb, Sr, V, and Zn were calibrated using Agilent ICP-MS Multi-element Calibration Standard 2A. Ti was calibrated using Agilent Multi-element Calibration Standard 4. Hg was calibrated separately using a single-element Agilent Hg standard. Working calibration standards were prepared by serial dilution of the stock standards in 2% *v*/*v* HNO_3_. Calibration blanks were prepared in the same matrix.

Matrix effects were minimized by matching the acid matrix of standards, blanks, and samples and further diluting digested samples when required to keep element concentrations within the linear calibration range. Element-specific limits of detection (LOD) and limits of quantification (LOQ) were established for the analytical method and are reported in Supplementary Table S1. All individual concentrations included in the statistical analyses were above their corresponding LOQ values; therefore, no substitution or censoring procedure for below-LOQ data was required.

Quality assurance and quality control procedures included reagent blanks, procedural blanks, calibration blanks, duplicate digestions, and the carbonate reference material MACS-3 (Microanalytical Carbonate Standard 3; United States Geological Survey, Denver, CO, USA). Recoveries for selected certified or reference elements available in MACS-3, including Al, As, Ba, Cd, Co, Cr, Cu, Fe, Mg, Mn, Ni, Pb, Sr, V, and Zn, ranged from 97 to 102%. Precision was evaluated through duplicate digestions included in each analytical batch. Hg quality control included separate Hg calibration, monitoring of Hg in calibration and procedural blanks, and verification that Hg signals in samples were above blank levels before interpretation. Calibration coefficients were higher than r^2^ = 0.997. All results are expressed as mg/kg air-dried weight.

### 2.3. Data Analysis

The raw data used for the statistical analyses are provided as Supplementary Data S1. All statistical analyses were performed in R (v4.4.1) using the packages dplyr (v1.1.4), tidyr (v1.3.1), purrr (v1.1.0), ggplot2 (v4.0.0), ggrepel (v0.9.6), flextable (v0.9.10), officer (v0.7.0), and stringr (v1.5.1). Data were first screened for missing values, non-positive values, and basic distributional properties.

#### 2.3.1. Comparative Analyses Among Genera

To assess intergeneric differences in elemental concentrations, a Kruskal–Wallis test was applied to each element, followed by Dunn’s post hoc pairwise comparisons with Bonferroni-adjusted *p*-values. Genera were grouped using significance letters derived from multcompView (v0.1.10). For each element and genus, median and interquartile range (IQR) values were calculated.

#### 2.3.2. Internal Residual Enrichment Factor (EF) Analysis

Because no independent local geochemical background was available for the sampled shell accumulation site, EF values were calculated as internal residual enrichment factors rather than as conventional enrichment factors relative to an external sedimentary or crustal background. The aim of this approach was to evaluate relative deviations from the lithogenic relationship within the shell dataset. The lithogenic reference element was selected by comparing the mean Spearman correlation coefficient (ρ) of Al and Ti with a set of terrigenous tracers (Fe, V, Cr, and Ni). The element with the highest mean correlation was adopted as the internal lithogenic reference element for residual EF calculation. Residual EFs were then estimated for each element, excluding the selected reference element and Ca, using log–log linear regressions against the chosen reference element. For each element, the expected lithogenic component was predicted from the fitted model, and the residual EF was calculated as the ratio between observed and predicted concentrations. Median residual EF values were then summarized by genus. Values above 1 were interpreted only as relative positive deviations from the modeled Al-based lithogenic expectation within the present shell dataset, not as evidence of anthropogenic enrichment or environmental contamination.

#### 2.3.3. Relative Concentration Factor (RCF) and Relative Multielement Loading Index (MPI)

The RCFs were calculated for each element as the ratio between the concentration measured in each shell and the dataset-wide median concentration of that element. This approach was used to compare relative element-specific deviations among genera within the present shell dataset. Because the denominator was an internal dataset median rather than an external environmental or geochemical background value, RCF should not be interpreted as a conventional contamination factor or as direct evidence of environmental contamination. Ca was excluded from the RCF calculation because it represents the dominant carbonate matrix component. Prior to calculation, non-positive values were treated as missing; however, no missing, zero, negative, non-finite, or censored values were detected in the element concentration data.

The MPI was calculated for each shell as the geometric mean of the positive concentrations of the included elements, excluding Ca. In this study, the MPI was used as a relative multielement loading index summarizing multielement loading within the dataset, and not as a direct measure of environmental pollution. Median RCF and MPI values were then summarized by genus.

#### 2.3.4. Multivariate Analysis

To explore multielement relationships, principal component analysis (PCA) was performed on centered log-ratio (clr)-transformed concentration data, excluding Ca, in order to account for the compositional nature of the dataset. The elements included in the PCA were Al, As, Ba, Cd, Co, Cr, Cu, Fe, Hg, Mg, Mn, Ni, Pb, Sr, Ti, V, and Zn. Prior to clr transformation, the data matrix was checked for missing, zero, negative, non-finite, and below-LOQ values. No such cases were detected among the concentrations included in the PCA; therefore, no zero replacement, censoring procedure, or median imputation was applied. PCA was conducted on scaled clr-transformed variables. The resulting biplot was used to visualize genus-level clustering and elemental loadings, and 95% confidence ellipses were calculated for each genus group from the corresponding covariance matrices.

## 3. Results

### 3.1. Descriptive Concentrations and Intergeneric Differences

The median concentrations and interquartile ranges (IQRs) of the elements measured in the shells of the five bivalve genera are presented in Table 1. Median elemental concentrations varied over several orders of magnitude. Ca was the dominant element in all genera, followed by Mg and, in most genera, by Al and Fe. Zn, Sr, Mn, and Ba showed intermediate median concentrations, whereas Hg and Cd were consistently among the lowest-concentration elements. Other trace elements, including As, Pb, Ni, Cu, Cr, and V, generally occurred at lower concentrations than the carbonate- and lithogenic-associated elements, although marked genus-specific exceptions were observed, particularly for Co in *Hypanis* and *Mytilaster*. The Kruskal–Wallis test revealed significant intergeneric differences (*p* < 0.05) for most elements, indicating that shell elemental composition varied markedly among genera.

Among the lithogenic-related elements, Al and Fe reached their highest median concentrations in *Dreissena*, followed by *Cerastoderma* and *Didacna*, whereas *Hypanis* and *Mytilaster* showed the lowest median values. Median Ca concentrations also varied among genera, with the highest values in *Dreissena* and the lowest in *Mytilaster*. Among the trace elements, median Cu, Zn, and Cd concentrations showed marked variation among genera. *Hypanis* exhibited the highest median Cu concentrations, whereas *Dreissena* showed the highest median Zn and Cd concentrations. In contrast, *Mytilaster* and *Didacna* generally presented lower median concentrations of these elements. Other elements, including Cr, Ni, and V, also varied significantly among genera, with the highest median concentrations generally observed in *Dreissena* and the lowest in *Mytilaster*. Median Hg and Pb concentrations remained low across all genera and showed less pronounced variation, although *Cerastoderma* displayed slightly higher median Hg concentrations and *Dreissena* slightly higher median Pb concentrations.

A particularly marked contrast was observed for Al, As, and Co. *Hypanis* and *Mytilaster* showed very low median Al and As concentrations compared with *Cerastoderma*, *Didacna*, and *Dreissena*, whereas the opposite pattern was observed for Co, with *Hypanis* and *Mytilaster* showing the highest median values. This pattern was not associated with missing, zero, negative, non-finite, or below-LOQ values in the analytical dataset. Therefore, it was retained as an element-specific genus-related pattern within the present dataset, although its mechanistic interpretation requires caution.

### 3.2. Lithogenic Reference Selection and Residual EFs

**Figure 2:**
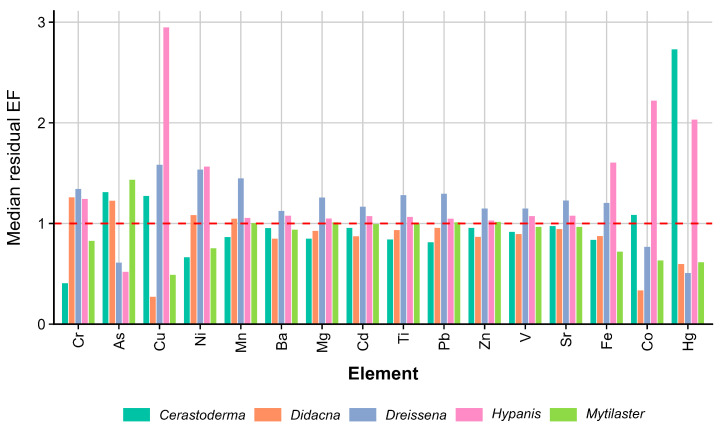
Median residual enrichment factors (EFs) of elements in bivalve shells from the Caspian Sea, calculated using Al as the internal lithogenic reference element. The dashed red line indicates EF = 1, corresponding to the modeled Al-based lithogenic expectation within the shell dataset.

Al was selected as the internal lithogenic reference element because it showed a stronger mean Spearman correlation with the terrigenous tracer set (Fe, V, Cr, and Ni) than Ti (mean ρ = 0.693 for Al versus 0.558 for Ti). Median residual EF values showed clear variation among both elements and genera (Figure 2). These EF values should therefore be interpreted as internal residual deviations from the Al-based lithogenic relationship within the present shell dataset, rather than as enrichment relative to an independent external geochemical background or as evidence of anthropogenic contamination.

To provide a more objective description of the EF patterns, median EF values were interpreted descriptively as slight relative enrichment when values were between 1.0 and 1.5, moderate relative enrichment when values were between 1.5 and 2.0, and pronounced relative enrichment when values were ≥2.0. At the genus level, *Hypanis* showed pronounced relative enrichment for Cu (2.95), Co (2.22), and Hg (2.03), together with moderate relative enrichment for Fe (1.60) and Ni (1.56). *Cerastoderma* showed pronounced relative enrichment for Hg (2.73) and slight relative enrichment for As (1.31), Cu (1.27), and Co (1.08). *Dreissena* displayed the broadest pattern of slight-to-moderate relative enrichment, with median EF values above 1 for Cu (1.58), Ni (1.54), Mn (1.45), Cr (1.34), Pb (1.30), Ti (1.28), Sr (1.23), Fe (1.20), V (1.15), Zn (1.15), and Ba (1.13). *Didacna* showed slight relative enrichment for Cr (1.26), As (1.21), Ni (1.08), and Mn (1.05), with most other elements near 1 or lower. *Mytilaster* showed median EF values close to 1 for most elements, with slight relative enrichment only for As (1.43). These results indicate genus-specific deviations from the Al-based lithogenic relationship, with pronounced element-specific patterns in *Hypanis* and *Cerastoderma* and a broader but mostly slight-to-moderate pattern in *Dreissena*.

### 3.3. Relative Concentration Factor (RCF) and Relative Multielement Loading Index (MPI)

Median relative RCF values showed notable variation among both elements and genera (Table 2). In most cases, median RCF values were close to 1, indicating concentrations near the dataset-wide median concentration for each element. However, marked relative increases were observed for several element–genus combinations. The highest median RCF values were recorded for Co in *Hypanis* (11.2) and *Mytilaster* (7.94), followed by Hg in *Cerastoderma* (4.85), Cu in *Hypanis* (3.78), and Cr in *Dreissena* (3.61) and *Didacna* (2.70). These patterns indicate marked genus-related differences in the relative shell concentrations of specific elements. In contrast, *Hypanis* and *Mytilaster* showed very low median RCF values for Al and As (≤0.02), whereas *Dreissena* exhibited median RCF values above 1 for most included elements, including Al, Ba, Cd, Cr, Cu, Fe, Mg, Mn, Ni, Pb, Sr, Ti, V, and Zn. This indicates a broadly elevated relative concentration pattern in *Dreissena* compared with the dataset-wide median for each element.

MPI values also differed among genera. The highest median MPI values were recorded in *Cerastoderma* (27.6) and *Dreissena* (27.1), followed by *Didacna* (19.3). Lower median values were found in *Hypanis* (16.6), whereas *Mytilaster* showed the lowest median MPI (10.2). In the context of this study, MPI should be interpreted as a comparative index of relative multielement loading within the shell dataset, rather than as a direct measure of environmental pollution. Accordingly, these results indicate that *Cerastoderma* and *Dreissena* had the highest relative multielement loading within the present dataset, whereas *Mytilaster* had the lowest among the genera analyzed.

### 3.4. Multivariate Analysis of Shell Elemental Composition

**Figure 3:**
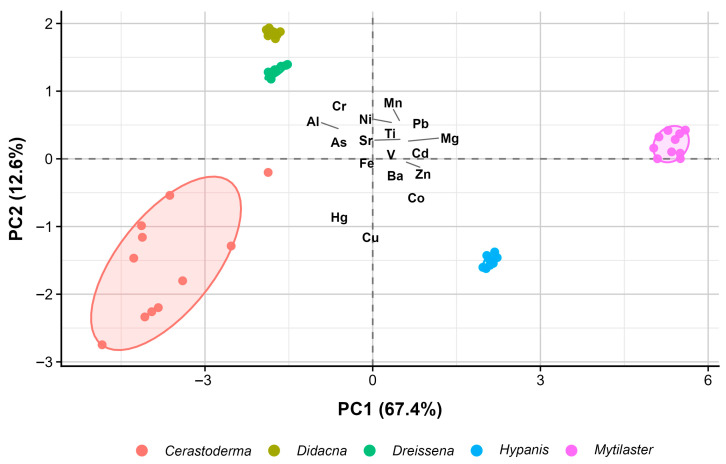
Multivariate separation of bivalve genera according to shell metal composition. PC1 and PC2 together explain 80% of the total variance; ellipses show the 95% confidence area for each group.

The PCA performed on clr-transformed elemental concentrations showed that the first two principal components explained 80.0% of the total variance (PC1 = 67.4%, PC2 = 12.6%). The biplot (Figure 3) revealed a structured distribution of bivalve genera according to shell elemental composition. PC1 represented the main axis of variation and was positively associated with Ba, Cd, Mg, Mn, Ni, Pb, Sr, Ti, V, and Zn and negatively associated with Al, As, Cr, and Fe. PC2 was mainly defined by negative loadings of Co, Cu, and Hg and positive loadings of Cr, indicating a secondary axis of variation related to these elements. In the ordination space, *Cerastoderma* was positioned in the negative quadrant of both components, in association with Al and As. *Dreissena* and *Didacna* were located in the negative PC1/positive PC2 quadrant, where Cr, Fe, and Mn showed stronger contributions. *Hypanis* occupied the positive PC1/negative PC2 sector and was associated mainly with Cu and Co, whereas *Mytilaster* was positioned toward the positive end of PC1, in association with Ba, Mg, Pb, Sr, and Ti.

## 4. Discussion

The abundance pattern, dominated by Ca and Mg, which are typical of carbonate matrices, together with appreciable contributions of Al and Fe, is consistent with shell composition being strongly influenced by the carbonate matrix and by additional non-carbonate components. These components may include lithogenic or surface-associated particles, such as Fe–Mn oxides and fine terrigenous material, either adhering to the shell surface or incorporated during shell formation [15]. The significant differences among genera detected by the Kruskal–Wallis test, particularly for Al, Fe, Cu, Zn, and Cd, further indicate that shell chemistry does not simply reflect a uniform bulk environmental signal but varies among taxa in a way that may involve both external element availability and genus-dependent incorporation processes [16,17].

The higher Al and Fe contents observed in *Dreissena*, compared with the lower values in *Hypanis* and *Mytilaster*, indicate clear genus-related differences in the contribution of lithogenic- or particle-associated elements to the shell material. However, the mechanisms behind these differences cannot be resolved from the present dataset alone. They may reflect differences in shell mineralogy, microstructure, exposure to fine particles, or genus-dependent incorporation processes, but these possibilities remain hypotheses in the absence of paired environmental, mineralogical, and soft-tissue data [18,19]. Previous biomonitoring studies have shown that bivalves may interact strongly with dissolved and particulate metal pools and that taxon-specific traits can influence metal incorporation [20,21]. Nevertheless, in the present study, such mechanisms can only be proposed as possible explanations rather than demonstrated causes. This caution is particularly important for the marked contrast observed in *Hypanis* and *Mytilaster*, where very low Al and As concentrations coincided with very high Co concentrations. This pattern may indicate genus-specific shell chemistry or biological incorporation, as bivalve shell microstructure and organic matrix composition can differ markedly among taxa even in apparently similar carbonate shell layers [22,23,24]. However, methodological factors, surface-associated phases, preservation history, postmortem alteration, or mineralogical differences cannot be excluded, because preservation state and diagenetic modification can affect the geochemical composition of mollusk shell carbonate [25].

The residual EF analysis, calculated using Al as the internal lithogenic reference element, provides a comparative way to identify element-specific deviations from the Al-based relationship within the shell dataset. Al was selected because it showed stronger internal correlations with terrigenous tracers than Ti; however, this normalization does not represent an independent geochemical background. Therefore, residual EF values above 1 should be interpreted as relative positive deviations from the modeled Al-normalized relationship, rather than as direct evidence of environmental enrichment, bioavailability, or anthropogenic inputs. Within this framework, the most distinctive positive deviations were observed for Cu, Co, and Hg in *Hypanis*, Hg in *Cerastoderma*, and a broader, slight-to-moderate pattern involving several elements in *Dreissena*. *Didacna* showed only slight positive deviations for Cr, As, Ni, and Mn, whereas *Mytilaster* remained close to the Al-based expectation for most elements. These patterns may be compatible with differences in shell mineralogy, surface-associated Fe–Mn oxides, organic complexes, or genus-dependent incorporation processes [26,27,28,29], but the present single-site dataset does not allow lithogenic, biological, diagenetic, and anthropogenic contributions to be separated. From a methodological perspective, the use of Al as a normalizer is appropriate for this internal comparative analysis, although comparison with Ti or other conservative reference elements may be useful in future studies, particularly where co-precipitation, adsorption, or reactive mineral phases are expected [30].

The RCF values further highlight the heterogeneous distribution of individual elements among genera, with particularly high median values for Co in *Hypanis* and *Mytilaster*, Hg in *Cerastoderma*, Cu in *Hypanis*, and Cr in *Dreissena* and *Didacna*. In contrast to RCF, which expresses element-specific deviations with respect to the dataset-wide median, MPI summarizes relative multielement loading across the elements included in the calculation. According to this index, the ranking of relative multielement loading was *Cerastoderma* ≈ *Dreissena* > *Didacna* > *Hypanis* > *Mytilaster*, in agreement with the broader patterns observed in the concentration data and RCF results [31,32,33]. These results indicate that *Cerastoderma* and *Dreissena* showed the highest overall relative multielement loading within the present dataset, whereas *Mytilaster* showed the lowest MPI despite its marked element-specific Co increase. Therefore, RCF and MPI should be interpreted as complementary internal metrics: RCF identifies specific element–genus contrasts, while MPI provides a comparative summary of multielement loading. Neither index should be interpreted as a direct measure of environmental contamination or as evidence of background conditions.

The PCA performed on clr-transformed data (PC1 = 67.4%; PC2 = 12.6%) confirmed a structured separation of genera according to multielement shell composition. PC1 represented the main compositional gradient, with positive associations for Ba, Cd, Mg, Mn, Ni, Pb, Sr, Ti, V, and Zn and negative associations for Al, As, Cr, and Fe. Because PCA loadings describe covariance structure rather than sources, this gradient should not be interpreted as a direct separation between lithogenic and biologically controlled inputs. Instead, it indicates that these groups of elements varied in opposite directions within the shell dataset. PC2 was mainly defined by negative loadings of Co, Cu, and Hg and positive loadings of Cr, with additional contributions from Mn and Ni. The resulting grouping pattern—*Hypanis* (PC1+, PC2−; mainly Cu/Co/Hg), *Dreissena*–*Didacna* (PC1−, PC2+; mainly Cr/Fe/Mn), and *Mytilaster* (PC1+; mainly Ba/Mg/Pb/Sr/Ti)—is consistent with previous studies indicating that shell geochemistry may integrate both environmental and biologically mediated signals [34]. However, in the present study, the PCA should be interpreted as an exploratory description of compositional relationships among genera and elements, rather than as evidence of specific lithogenic, biological, or anthropogenic sources.

Taken together, the results show that Caspian bivalve shells display distinct genus-related multielement profiles that can be consistently described using median concentrations, residual EF, RCF, MPI, and PCA-clr analysis. This is the main value of the study: even within a single shell accumulation site, shell chemistry was not random but showed structured differences among genera. These differences provide a useful comparative framework for identifying genera and elements that may deserve priority in future biomonitoring-oriented studies. In particular, *Dreissena* and *Cerastoderma* showed the highest relative multielement loading, whereas *Hypanis* displayed marked element-specific deviations for Cu, Co, and Hg. *Mytilaster* and *Didacna* generally showed lower overall multielement loading, although *Mytilaster* also exhibited a strong Co-specific pattern. Therefore, the dataset does not simply identify “high” and “low” metal genera but highlights complementary genus-specific patterns that may be useful for selecting target taxa, elements, and hypotheses in future monitoring designs. This interpretation is consistent with previous studies indicating that shell geochemistry can reflect a combination of environmental availability, particulate interactions, shell structure, and biologically mediated incorporation processes [9,10,34]. Accordingly, the present study provides an initial comparative basis for using bivalve shells as complementary archives of multielement shell signatures in the Caspian Sea. Future work integrating dissolved, particulate, sedimentary, mineralogical, and soft-tissue data, together with living populations and broader spatial replication, will be necessary to test source attribution and to evaluate the monitoring value of individual genera across coastal environments.

The main limitation of this study is that all shells were obtained from a single shell accumulation site rather than from standardized sampling of living populations across multiple locations. Although only adult, visibly well-preserved shells without visible abrasion were selected, and specimens with comparable external growth-ring patterns were preferentially included within each genus, differences in postmortem history, residence time in the accumulation deposit, preservation state, previous exposure conditions, and chronological age cannot be completely excluded. This selection was intended to reduce, although not eliminate, potential ontogenetic or size-related effects. Exploratory checks based on shell size, shell weight, and external growth-ring counts did not indicate that these variables alone accounted for the main genus-level multielement patterns observed in the dataset. Nevertheless, because the exact age could not be determined for the accumulated shells, residual size- or age-related effects cannot be fully excluded. In addition, the absence of paired sediment, water, suspended particulate matter, and soft-tissue data prevents direct source attribution and limits the ability to separate lithogenic, biological, diagenetic, and anthropogenic contributions. The lack of mineralogical, microstructural, and controlled cleaning or leaching analyses also limits the interpretation of element-specific contrasts, particularly for elements showing unusual genus-specific patterns such as Co. Therefore, the present results should be interpreted as a preliminary within-dataset comparison of genus-related shell metal profiles, rather than as definitive evidence of spatial pollution patterns or direct environmental contamination levels in the southern Caspian Sea. Despite these limitations, the study identifies structured shell metal signatures that can guide future biomonitoring-oriented research based on replicated sampling, living populations, paired environmental compartments, and shell mineralogical characterization.

## 5. Conclusions

The multielement assessment of bivalve shells from the southern Caspian Sea showed clear genus-related differences in shell metal profiles within the studied shell accumulation deposit. *Dreissena* and *Cerastoderma* displayed the highest relative multielement loading, whereas *Mytilaster* and *Didacna* generally showed lower overall loading; *Hypanis* was characterized by marked element-specific deviations, particularly for Cu, Co, and Hg. The combined use of median concentrations, internal residual EF, RCF, MPI, and PCA-clr analysis provided a coherent comparative framework for describing these genus-related patterns and identifying elements and taxa that may deserve priority in future biomonitoring-oriented studies.

However, these findings should be interpreted within the limits of the study design. Because the dataset was based on empty shells from a single accumulation site and lacked paired sediment, water, suspended particulate matter, soft-tissue, mineralogical, and microstructural data, the observed shell profiles cannot be used to assign specific lithogenic, biological, diagenetic, or anthropogenic sources nor to infer direct environmental contamination levels. Thus, the present study does not validate these genera as definitive monitoring indicators but provides a preliminary comparative basis for evaluating the potential of Caspian bivalve shells as complementary archives of multielement shell signatures. Future work should include replicated spatial sampling, living size- or age-classed populations, standardized cleaning or leaching tests, shell mineralogical characterization, and integration with environmental and tissue compartments to test source attribution and monitoring applicability.

## Figures and Tables

**Table 1:** Median concentrations (IQR) of elements measured in bivalve shells.

Metal(oid) (mg/kg)	*Cerastoderma*	*Didacna*	*Dreissena*	*Hypanis*	*Mytilaster*
Al	6033 (5350–6568) ^a^	5313 (5243–5369) ^ab^	7262 (7119–7412) ^a^	112 (108–115) ^bc^	38.2 (32.2–42.8) ^c^
As	10.3 (10.2–16.2) ^a^	9.41 (9.31–9.94) ^ab^	6.36 (6.20–6.51) ^bc^	0.11 (0.11–0.11) ^c^	0.12 (0.11–0.13) ^c^
Ba	65.0 (58.3–70.8) ^ab^	57.6 (56.1–60.9) ^ac^	77.44 (75.92–80.28) ^b^	62.0 (60.6–62.4) ^abc^	51.4 (50.6–57.2) ^c^
Ca	308,162 (278,715–347,102) ^ab^	270,372 (263,721–290,018) ^ac^	398,732 (389,575–447,986) ^b^	287,156 (285,830–296,084) ^abc^	262,232 (216,653–271,740) ^c^
Cd	0.10 (0.09–0.11) ^ab^	0.09 (0.09–0.10) ^a^	0.12 (0.12–0.13) ^b^	0.10 (0.09–0.10) ^ab^	0.09 (0.08–0.09) ^a^
Co	6.66 (5.42–88.4) ^ab^	2.92 (2.75–3.07) ^a^	5.05 (4.96–5.09) ^ab^	497 (471–509) ^c^	354 (317–371) ^bc^
Cr	34.4 (18.9–95.9) ^ab^	75.0 (70.9–77.7) ^ab^	100 (99.4–101) ^a^	3.94 (3.73–4.02) ^bc^	1.19 (1.13–1.27) ^c^
Cu	5.03 (3.63–9.04) ^ab^	1.09 (1.05–1.11) ^c^	6.10 (5.82–6.29) ^ab^	18.2 (17.7–18.5) a	3.49 (3.14–3.75) ^bc^
Fe	133 (122–158) ^a^	137 (131–143) ^ab^	197 (196–201) ^b^	130 (127–136) ^ac^	48.4 (45.9–51.9) ^c^
Hg	1.32 (0.73–1.64) ^a^	0.27 (0.27–0.28) ^b^	0.25 (0.24–0.25) ^bc^	0.37 (0.36–0.39) ^ab^	0.08 (0.08–0.09) ^c^
Mg	596 (482–739) ^a^	651 (630–662) ^a^	890 (829–898) ^b^	667 (656–690) ^ab^	626 (567–656) ^a^
Mn	71.8 (29.0–93.2) ^a^	87.0 (85.3–90.1) ^a^	120 (114–124) ^b^	88.4 (87.2–91.4) ^ab^	84.4 (79.7–88.2) ^a^
Ni	4.59 (3.17–5.76) ^a^	7.52 (7.39–7.67) ^ab^	10.8 (10.1–11.2) ^b^	9.10 (8.65–9.11) ^b^	4.16 (3.65–4.75) ^a^
Pb	1.85 (1.50–2.32) ^a^	2.18 (2.11–2.25) ^a^	2.96 (2.83–3.10) ^b^	2.24 (2.14–2.25) ^a^	2.12 (1.89–2.24) ^a^
Sr	6.41 (3.85–6.80) ^a^	6.21 (6.05–6.35) ^a^	8.15 (8.02–8.26) ^b^	6.42 (5.91–6.65) ^ab^	5.62 (5.46–6.02) ^a^
Ti	316 (242–384) ^a^	352 (342–382) ^a^	486 (456–492) ^b^	371 (358–377) ^a^	338 (300–372) ^a^
V	10.6 (9.39–12.9) ^ab^	10.3 (9.66–10.5) ^a^	13.4 (12.8–13.9) ^b^	10.5 (10.2–10.8) ^ab^	9.12 (8.48–9.92) ^a^
Zn	161 (145–174) ^ab^	146 (141–147) ^a^	195 (187–196) ^b^	154 (146–159) ^a^	146 (140–155) ^a^

^abc^ Different superscript letters within the same row indicate significant differences among genera according to Kruskal–Wallis tests followed by Dunn’s post hoc comparisons with Bonferroni adjustment (*p* < 0.05). Genera sharing at least one letter are not significantly different.

**Table 2:** Median relative concentration factor (RCF) values by element and median relative multielement loading index (MPI) values by genus. RCF values > 1 indicate relative enrichment with respect to the dataset-wide median concentration of each element.

Metal	*Cerastoderma*	*Didacna*	*Dreissena*	*Hypanis*	*Mytilaster*
Al	1.15	1.01	1.38	0.02	0.01
As	1.63	1.47	1.00	0.02	0.02
Ba	1.06	0.94	1.26	1.01	0.84
Cd	1.04	0.95	1.29	1.00	0.89
Co	0.15	0.07	0.11	11.2	7.94
Cr	1.24	2.70	3.61	0.14	0.04
Cu	1.04	0.23	1.26	3.78	0.72
Fe	1.01	1.04	1.50	0.98	0.37
Hg	4.85	1.00	0.91	1.37	0.31
Mg	0.90	0.98	1.34	1.01	0.94
Mn	0.82	0.99	1.37	1.01	0.96
Ni	0.62	1.01	1.45	1.23	0.56
Pb	0.83	0.98	1.33	1.00	0.95
Sr	1.01	0.98	1.29	1.02	0.89
Ti	0.87	0.96	1.33	1.01	0.93
V	1.01	0.98	1.27	1.00	0.87
Zn	1.04	0.95	1.26	1.00	0.94
MPI	27.6	19.3	27.1	16.6	10.2

## Data Availability

Data are contained within the Supplementary Material.

## Supplementary Materials

The following supporting information can be downloaded at: https://www.mdpi.com/article/10.3390/ani16101491/s1, Supplementary Table S1: Element-specific method limits of detection (LOD) and quantification (LOQ) for ICP-MS analysis of bivalve shell samples; Supplementary Data S1: dataset supporting the results presented in this study.

## Abbreviations

The following abbreviations are used in this manuscript:

ICP-MS	Inductively Coupled Plasma–Mass Spectrometry
EF	Internal residual enrichment factor
RCF	Relative concentration factor
MPI	Relative multielement loading index
PCA-clr	Principal component analysis on centered log-ratio-transformed data
PC	Principal component
IQR	Interquartile range
