# Supplementary material for: Shell Metal Profiles of Caspian Bivalves Show Genus-Specific Patterns with Potential Relevance for Biomonitoring in the Southern Caspian Sea
Source: Animals (Basel). 2026 May 13;16(10):1491. doi: 10.3390/ani16101491 (PMC13203848; doi:10.3390/ani16101491)
Supplement: Supplementary file 1 [file animals-16-01491-s001.zip › Supplementary Table S1.pdf]

Supplementary Table S1. Element-specific method limits of detection (LOD) and quantification (LOQ) for ICP-MS analysis of bivalve shell samples.

Element	LOD	LOQ	Units
Al	0.089	0.271	mg/kg
As	0.007	0.021	mg/kg
Ba	0.012	0.037	mg/kg
Ca	0.081	0.244	mg/kg
Cd	0.002	0.007	mg/kg
Co	0.003	0.008	mg/kg
Cr	0.003	0.009	mg/kg
Cu	0.003	0.008	mg/kg
Fe	0.082	0.248	mg/kg
Hg	0.002	0.006	mg/kg
Mg	0.222	0.673	mg/kg
Mn	0.084	0.256	mg/kg
Ni	0.023	0.070	mg/kg
Pb	0.009	0.028	mg/kg
Sr	0.003	0.010	mg/kg
Ti	0.016	0.048	mg/kg
V	0.157	0.477	mg/kg
Zn	0.224	0.678	mg/kg

Values are expressed as mg/kg dry weight. All individual concentrations included in the statistical analyses were above their corresponding LOQ values.
